# Pre-treatment loss to follow-up among children with multidrug-resistant tuberculosis in South Africa, 2008–2010

**DOI:** 10.1371/journal.pone.0230504

**Published:** 2020-04-07

**Authors:** Brittany K. Moore, Linda Erasmus, Julia Ershova, Sarah E. Smith, Norbert Ndjeka, Laura J. Podewils

**Affiliations:** 1 U.S. Centers for Disease Control and Prevention, Atlanta, Georgia United States of America; 2 National Institute for Communicable Diseases, National Health Laboratory Service, Johannesburg, South Africa; 3 South Africa National Department of Health, Pretoria, South Africa; University of KwaZulu-Natal, SOUTH AFRICA

## Abstract

Multidrug-resistant (MDR) TB is more difficult to diagnose and treat compared with drug-susceptible TB. Young children are at greater risk of severe TB disease and death when treatment is delayed compared to adults. We sought to describe characteristics of children (<13 years) diagnosed with MDR TB between 2008–2010 in three South African provinces and assess factors associated with pre-treatment loss to follow-up. We matched laboratory and medical records at treatment facilities to identify pre-treatment loss and examined demographic and clinical characteristics for association with loss. Categorical variables were examined for association using Pearson’s *x*^2^ or Fisher’s exact test, employing Bonferroni correction for multiple pairwise comparisons. Between 2008–2010, 156 children were diagnosed with laboratory-confirmed MDR TB. Only 44% (n = 69) were documented as having received treatment. Young children (<2 years) (47/59, 80%), children with extrapulmonary (EP) TB (27/34, 79%), and children diagnosed at general hospitals (60/97, 62%) were most likely to be lost before treatment. Children most vulnerable to death from TB are most likely to be lost before treatment, possibly leading to underestimates of disease burden, case notifications, and poor outcomes among this population. Point-of-care diagnosis and robust follow-up may reduce pre-treatment loss in this population.

## Introduction

Each year, an estimated 10 million people become sick with active tuberculosis (TB) disease, leading to more than 1.45 million deaths.[1] The World Health Organization (WHO) estimates that more than one million of these cases and at least 200,000 deaths are among children (<15 years).[1] Recent estimates suggest that TB ranks as the sixth to ninth leading cause of death among children worldwide.[2,3] Vulnerability of young children to infection and disease after TB exposure has been well described.[3–8] However, historically, young children were not prioritized by TB programs due to the mistaken belief that they did not transmit TB and were at lower risk of mortality.[6,7,9] Bacteriologic confirmation of TB among children is difficult as young children often present with general complaints common to other childhood illnesses, cannot easily produce sputum for testing, and have paucibacillary or extrapulmonary disease more often than adults.[12] Such factors can result in delays in diagnosis, which is of particular concern for young children who are at greater risk of progressing rapidly from infection to disease and developing more severe, aggressive forms of TB. [9–12]

In 2018, there were an estimated 484,000 cases of rifampicin-resistant (RR) or multidrug-resistant (MDR) TB (strains of TB resistant to the two most effective anti-TB drugs, isoniazid and rifampicin) worldwide.[1] MDR TB is more difficult and costly to diagnose and treat than drug-susceptible TB and can have long-term side-effects, especially among young children (e.g. ototoxicity, psychological disorders, cartilage damage, thyroid dysfunction).[13–16] Modeling studies suggest there were between 25,000 to 32,000 cases of MDR TB among children each year, but these are believed to underestimate the true burden.[3,17,18] In 2018, 49% of children with TB were believed to have been undiagnosed and untreated; the proportion of children with drug-resistant TB who are undiagnosed or untreated is unknown.[1] Better understanding the limitations of existing surveillance systems and systematically quantifying the burden of TB and MDR TB among children is a priority of the global TB community.[9,10,17,19]

TB is the leading cause of death among adults in South Africa, and the fourth leading cause of death among children.[20] South Africa has the second highest TB incidence in Africa with 530,000 new TB cases each year.[21] In 2012, when these data were collected, South Africa reported 15,419 laboratory-confirmed cases of MDR TB with an estimated 1.8% of new cases and 6.7% of retreatment cases diagnosed with MDR TB.[21] The risk of developing MDR TB disease among children in South Africa is estimated to be higher than among adults given high rates of ongoing community-level transmission and unique vulnerability of young children to progression from infection to disease.[22] Previous studies have suggested underreporting of drug-susceptible and drug-resistant TB cases among adults and children in South Africa.[23–28]

We sought to evaluate the proportion of children (<13 years) diagnosed with MDR TB between 2008–2010 in three provinces of South Africa who were admitted for treatment at a designated MDR TB treatment facility and identify demographic and clinical factors associated with pre-treatment loss to follow-up.

## Materials and methods

This assessment included all children diagnosed with or treated for MDR TB in three South African provinces (Eastern Cape, Gauteng, and Limpopo) between 1 January 2008 and 30 June 2010. These provinces were purposively selected to represent a range of urban, peri-urban, and rural settings. During this time in South Africa, persons under 13 years of age were considered children, and official policy stated that treatment and clinical management of children with MDR TB only occurred at designated MDR TB units in tertiary level hospitals, often located in provincial capitals.[29,30] Children were assessed for having drug-resistant TB if they had a documented exposure to someone with drug-resistant TB, were being retreated for TB, or failed to respond to treatment with first-line drugs. Drug-resistant TB was diagnosed through drug susceptibility testing (DST) by liquid media (Mycobacteria Growth Indicator Tube) and Hain GenoType® MTBDR line probe assay (LPA) on culture positive specimens. [30] Confirmation of LPA results by phenotypic DST was conducted upon request. During the study period, MDR TB treatment in South Africa was only available at designated health facilities; bacteriologic confirmation of MDR TB was required for treatment for MDR TB. [29,30] There was limited treatment in specialist academic settings, mostly in Western Cape and Gauteng provinces, which did not always require laboratory confirmation for admission. Records from specialist academic settings treating pediatric TB patients in Gauteng Province were included in this analysis to ensure we captured all facilities at which this patient population could be treated. TB resistance categories were based on standard definitions for MDR TB and extensively drug-resistant (XDR) TB.[31] Because of the restrictions on treatment in place at the time, we believe that all children diagnosed with MDR TB would only have been treated at the included treatment facilities.

Several surveillance systems capture data on diagnosis and treatment of TB and drug-resistant TB in South Africa. South Africa’s National Health Laboratory Service (NHLS) routinely collects data on all patients with a specimen sent to any NHLS-affiliated laboratory for diagnosis of TB or drug-resistant TB through their Corporate Data Warehouse (CDW). During the study period, all specimens tested for drug resistance in the public sector throughout South Africa were processed through the NHLS laboratory network with results recorded in the CDW, with the exception of KwaZulu-Natal which housed its data elsewhere. Each MDR TB treatment facility maintains information on all admissions and treatment for patients with MDR TB. This analysis focuses on data available in the NHLS CDW and admissions records from the MDR TB units designated to treat children under 13 years of age in the three selected provinces. However, to ensure that we accurately captured record of treatment, we cross-referenced patient data with EDRWeb, a surveillance database of drug-resistant TB patients maintained by the National Department of Health, to determine if any child may have received care outside designated treatment facilities. If an individual was diagnosed in the public sector and treated in study provinces during the study period, that individual should be documented in all three systems captured in this analysis. Loss to follow-up was defined as any instance where a patient with a documented bacteriologic confirmation of MDR TB was not documented in facility admissions or treatment records or in EDRWeb.

We collected patient-level data from all three systems during the study period. These three systems operate independently, and patients cannot be automatically matched across these systems because they lack a common, unique identifier. To achieve a universal list of patients within and across these sources, data were matched using five criteria consistently available across all data sources: first name, surname, gender, TB registration year, and date of birth (DOB). We first applied deterministic algorithms using Microsoft Access® to obtain exact matches based on first name, surname and date of birth (DOB) or age. Second, a probabilistic matching procedure was applied to unmatched entries, designating as matched those entries with first name and surname within three letters and exact matches to two of three additional variables (registration year, DOB/age, and gender).[32] All automated matches were investigated manually for confirmation of match, and all remaining unmatched entries were manually compared, declaring a match if three or more criteria matched. The matching protocol was repeated by a second reviewer for data quality assurance.

Data were analyzed using the statistical analysis software R 3.5.1 (The R Foundation for Statistical Computing, Vienna, Austria). We calculated the number of unique individuals documented across any of the three data sources then calculated the proportion of individuals documented in each and any combination of data sources to assess completeness across systems. Demographic, diagnostic, and clinical characteristics of the study population were summarized using median and interquartile range (IQR) for age and frequencies of all categorical variables. The association between age as a continuous variable and failure to initiate treatment was evaluated using the Wilcox-Mann-Whitney test given violation of normality. The association between categorical sociodemographic and clinical variables and failure to initiate treatment was examined using the Pearson’s *x*^2^ test or Fisher’s exact test, where appropriate, with Bonferroni correction for multiple pairwise assumptions. An alpha level less than 0.05 was considered statistically significant.

Ethical approval was granted by the South Africa National Department of Health, Provincial Departments of Health, University of Witswatersrand, and the South African Medical Research Council. The study was reviewed in accordance with the U.S. Centers for Disease Control and Prevention (CDC) human research protection procedures and was determined to be a routine program evaluation. Data were anonymized after matching procedures were completed; informed consent was waived by the University of Witswatersrand upon determination that this was a routine program evaluation with appropriate data protection protocols.

## Results and discussion

### Database matching: Documentation of children diagnosed with MDR TB

**Fig 1:**
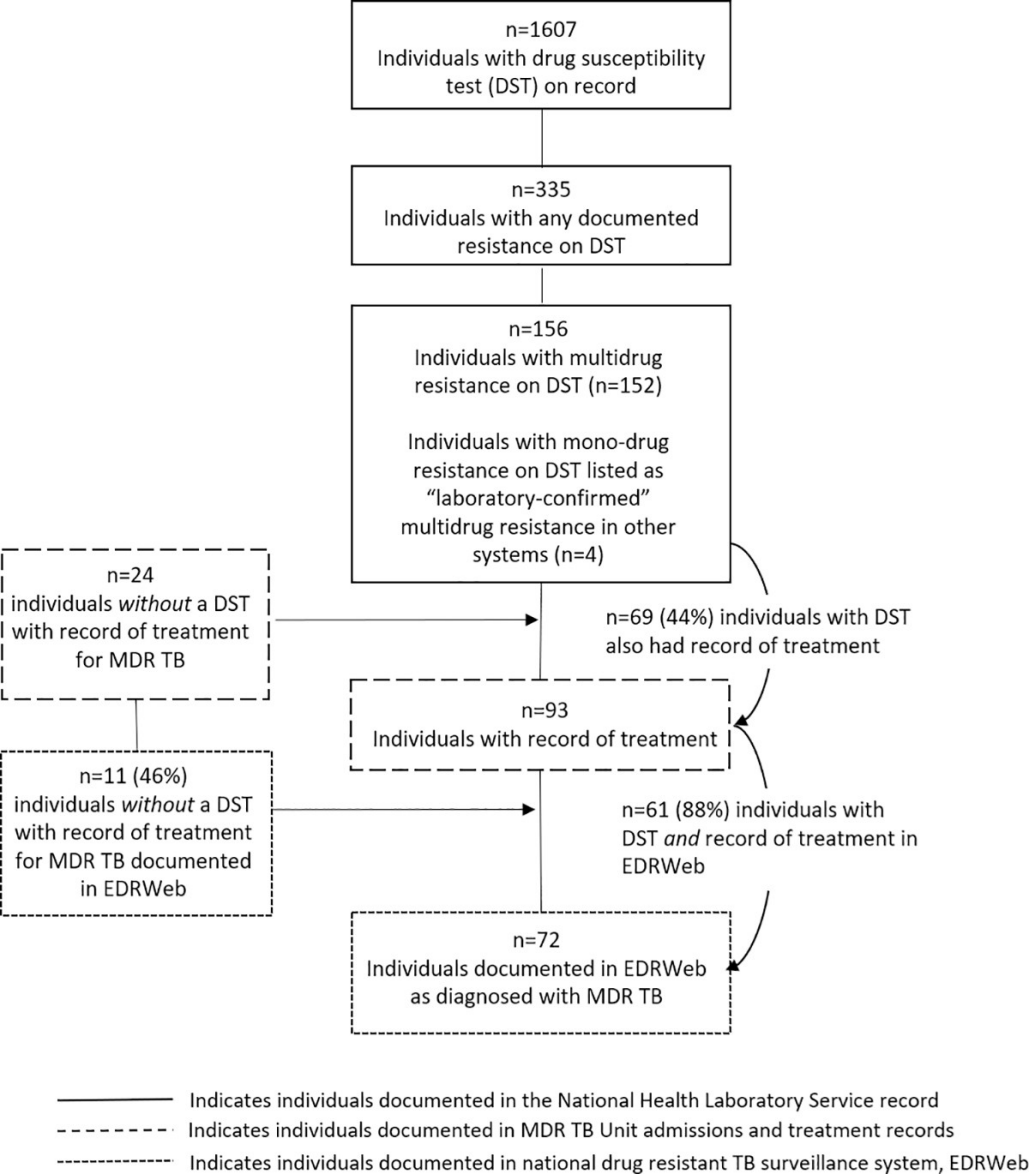
Diagnostic and clinical cascade of care for children (<13 years) with any documentation of MDR TB diagnosis as captured by laboratory, hospital, and national disease surveillance systems in three provinces in South Africa, 2008–2010.

**Fig 2:**
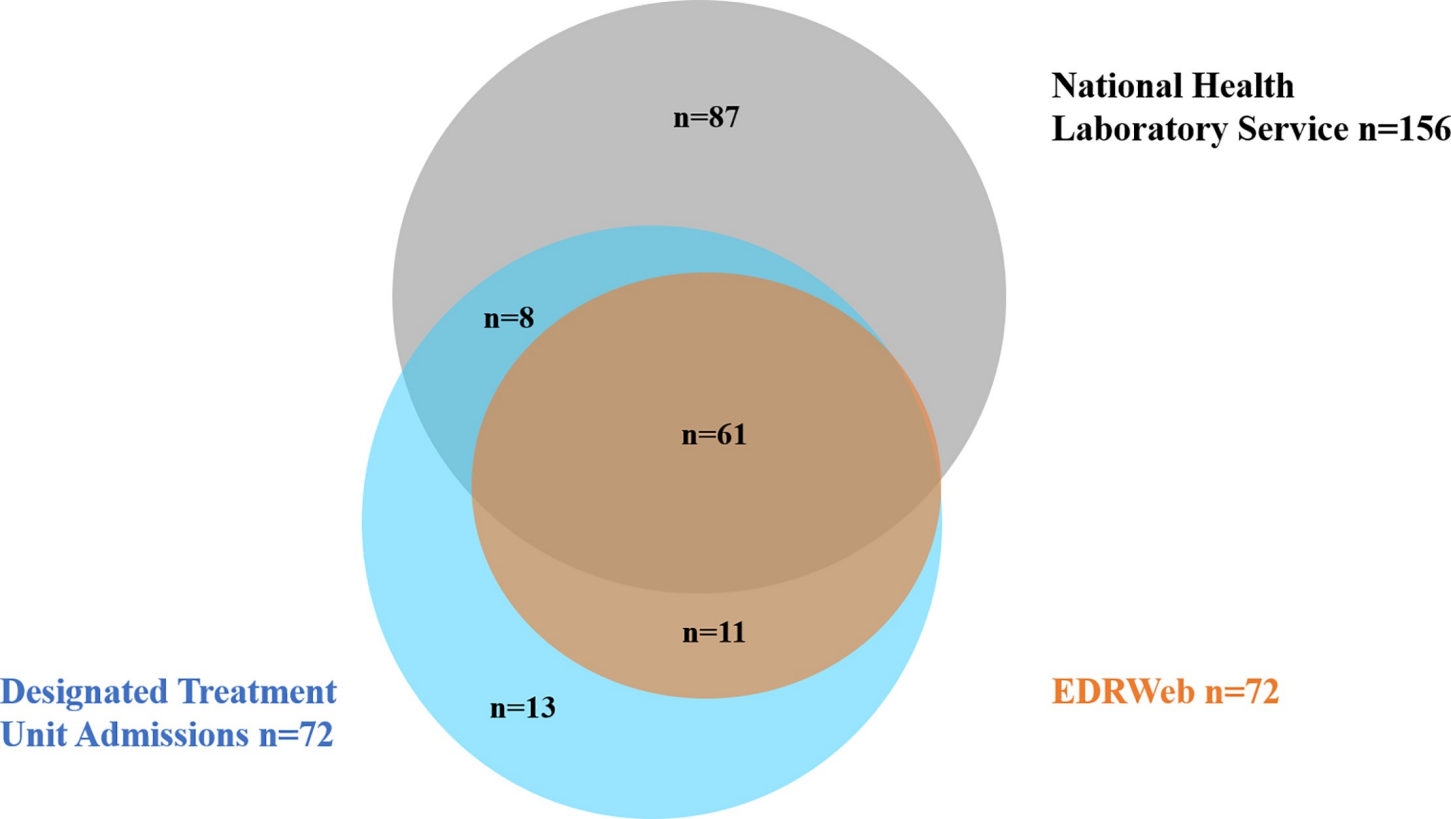
Children (<13 years) with MDR TB captured by laboratory, hospital, or disease surveillance systems (N = 180), in three provinces in South Africa, 2008–2010.

The CDW of NHLS captured results of both genotypic and phenotypic DST conducted for 1,607 individuals under 13 years of age between 1 January 2008 and 30 June 2010. Among those tested, 335 (21%) had documented resistance to any anti-TB drug, and 152 (9.5%) had documented MDR TB, of which 3 (<1%) had extensively drug-resistant (XDR) TB. There were a total of 180 unique individuals under 13 years of age documented in *any data source* as having MDR TB or XDR TB (Fig 1). There were four (2%) individuals documented in other data sources as having “laboratory-confirmed” MDR TB but their records at CDW indicated mono-drug resistance; these four individuals have been included in the analysis of laboratory data, but their documented drug resistance category from CDW is indicated (Table 1). There were 24 (13%) unique individuals documented as receiving treatment in admissions records (13) that did not have a laboratory-confirmed MDR TB diagnosis on record. These patients may have been treated based on clinical assessment and contact history despite the fact that clinical diagnosis *and treatment* of MDR TB was not common practice in these provinces at the time. All children found in EDRWeb had a documented admission record at a designated MDR TB treatment unit or academic hospital. Among all children with laboratory-confirmed MDR TB, only 69 (44%) were documented in any system as having received treatment and only 61 (39%) were documented in EDRWeb, the national surveillance system (Fig 2).

**Table 1:** Demographic and clinical characteristics of children with laboratory-confirmed drug-resistant TB in three provinces of south Africa 2008–2010 by record of treatment at designated treatment facilities (N = 156).

	Total (N = 156) n (%)	No Record of Treatment (n = 87) n(%)	Record of Treatment (n = 69) n(%)	P-value
Age (Median, IQR)^*^	6 [0–10]	2 [0–9]	9 [5–11]	p<0.001
**Gender**^†^	n = 150	n = 83	n = 67	p = 0.68
Female	80 (53)	43 (52)	37 (55)	
Male	70 (47)	40 (48)	30 (45)	
**Province**^‡^				p = 0.24
Eastern Cape	68 (44)	40 (46)	28 (41)	
Gauteng	78 (50)	44 (51)	34 (49)	
Limpopo	10 (6)	3 (3)	7 (10)	
**Level of Diagnosing Facility**^‡^				
Tertiary Care	18 (12)	1 (1)	17 (25)	Reference
General Hospital	97 (62)	60 (69)	37 (54)	P<0.001
Health Clinic	36 (23)	23 (26)	13 (19)	P<0.001
Community Health Center	5 (3)	3 (3)	2 (3)	p = 0.13
**Diagnostic Specimen Type**^†^				
Sputum	99 (64)	46 (53)	53 (78)	Reference
Aspirate	22 (14)	14 (16)	8 (12)	p = 0.67
Extrapulmonary Specimen	34 (22)	27 (31)	7 (10)	p = 0.01
**Drug Susceptibility Test Type**^†^				
LPA Only	26 (17)	20 (23)	6 (9)	Reference
MGIT Only	83 (53)	41 (47)	42 (61)	p = 0.08
LPA + MGIT	47 (30)	26 (30)	21 (30)	p = 0.34
**Drug Resistance Category**^‡^				
Multidrug Resistance	149 (96)	86 (99)	63 (91)	Reference
Monodrug Resistance	4 (3)	0 (0)	4 (6)	p = 0.10
Extensive Drug-Resistance	3 (2)	1 (1)	2 (3)	p = 1

*Wilcox-Mann-Whitney test for significance for Age

†Pearson’s Chi-sq test for significance for Gender, Drug Susceptibility Test Type, Specimen Type *(pairwise comparisons adjusted with Bonferroni method)*

‡Fisher’s Exact Test for significance for Province, Level of Diagnosing Facility, and Drug Resistance Category *(pairwise comparisons adjusted using Bonferroni method)*

### Diagnostic and clinical cascade among children with laboratory-confirmed MDR TB

Among the 156 children with laboratory-confirmed MDR TB, the median age was 6 years [IQR 0–10 years], more than half (53%) were female (n = 80/150, and half (n = 78, 50%) were from Gauteng (Table 1). Diagnostic work-up was most often performed at a general hospital (n = 97, 62%) using sputum specimens (n = 99, 64%). Diagnostic work-up using only LPA was performed for 26 (17%) children, while the rest received either LPA with confirmation by liquid media (n = 47, 30%) or liquid media alone (n = 83, 53%). Almost all (n = 152, 97%) of the children had a laboratory specimen on file with CDW with documented MDR TB or greater resistance. Four children had a laboratory specimen on file with CDW that indicated mono-drug resistance but they had medical and surveillance record documentation of MDR TB; their CDW results have been included in Table 1.

Among the 156 laboratory-confirmed MDR TB cases, 87 (56%) were not registered for treatment at a designated treatment facility. There was a significant difference in the median age among children with record of treatment compared to those with no such record (9 and 2 years, respectively; Mann–Whitney U = 1.819, P < 0.0001). Children diagnosed at a general hospital were less likely to have documentation of treatment (n = 37/97, 38%) compared to children diagnosed in any other settings (n = 32/59, 54%) (Table 1). There was also a statistically significant difference among the proportions of children registered for treatment by diagnostic specimen. Among 34 children diagnosed based on an extrapulmonary specimen, 27 (79%) were not registered for treatment compared with 53 (54%) of 99 children diagnosed with a sputum specimen. Young children (<5 years) were more likely to be diagnosed based on an aspirate or extrapulmonary specimen (39/66, 59%) compared with older children (16/90, 18%).

NHLS CDW records documenting DST among children during the study period revealed a higher proportion of isolates with any resistance (21%) and multidrug resistance (9.7%) compared with previous studies of culture-confirmed TB among children in Western Cape during the same time period (14.4%and 7.1%, respectively). Those studies suggested the epidemic among children was stabilizing.^12,23,24^ Our data may suggest higher rates of drug resistance among children in these provinces, though in the Western Cape study, all culture-confirmed isolates were tested for DST, while there may have been a higher index of suspicion of drug resistance among isolates tested for DST by NHLS in these study provinces.

While there were 156 laboratory-confirmed diagnoses of MDR TB among children in these three provinces, this is almost certainly an underestimate of the true burden considering the well-documented difficulties in bacteriologic confirmation in young children.[2,5,6,9]Our data indicate that even among children with a laboratory-confirmed diagnosis of MDR TB, more than half (56%) were lost to follow-up before receiving treatment. It is particularly concerning that very young children who are most likely to progress rapidly to severe forms of disease and are at highest risk of early mortality were the most likely to be lost to follow-up before treatment. Many of the youngest children had extrapulmonary or disseminated TB, a more severe form of disease also associated with higher mortality in very young children.[9–11,33] Children with confirmed or presumed extrapulmonary disease were more likely not to be registered for treatment than those with pulmonary disease.[33]

Only 39% (61) of laboratory-confirmed diagnoses were reflected in the national surveillance system capturing drug-resistant TB, suggesting the national program may underestimate case notifications and mortality before treatment and overestimate cure rates among children. This surveillance system is known to underestimate the true burden of MDR TB among adults, due in part to high rates of loss to follow up between diagnosis and treatment for adults with MDR TB (37%).[25,26] In addition, two studies evaluating recording and reporting of childhood TB at several facilities in Cape Town found that children with drug-resistant and disseminated or extrapulmonary TB were less likely to be reported through existing surveillance and laboratory systems than children with drug-susceptible TB.[27,28] The fact that many children lost to follow-up were evaluated in hospital or clinic settings suggests that there are limitations in referral mechanisms or results reporting at the laboratory or hospital and clinic settings leading to higher patient loss to follow-up. To our knowledge this is the first assessment of pre-treatment loss to follow-up among children diagnosed with drug-resistant TB.

Rapid diagnostics that can speed diagnosis of resistance among children are now in wide use across South Africa, and this may alleviate loss to follow-up by decreasing times associated with linkage to care. Recent revisions to policies removing the requirement for laboratory-confirmation of drug resistance prior to admission and treatment likely have enabled more rapid treatment initiation for children presumed to have drug-resistant TB.

### Limitations

This assessment relied on retrospective collection of programmatic data; our ability to describe true practice was limited by the accuracy and completeness of recorded data. Triangulation across data sources where error or incomplete entries may be common presents challenges for ensuring perfect fidelity of these data to individuals actually diagnosed and/or treated. During the study period, the practice of excluding children with mono-resistant or non-MDR poly-resistant TB from treatment at designated MDR TB Units limited our ability to describe other forms of drug-resistant TB. Format and completeness of variables differed by data source, province, and hospital, however, data were standardized across sources where possible. Because selected data sources are not sufficiently independent, we cannot estimate the true burden of MDR TB in the study provinces using the capture-recapture method, but believe these data accurately reflect case counts as documented by the relevant laboratory, hospital, and surveillance systems. It cannot be ruled out that some patient loss to follow-up could be a function of migration to other provinces for care, death, or failure to document patients at multiple steps in their care. We were unable to match data to mortality registers in South Africa given the lack of a national unique identifier as well as incomplete documentation of mortality, especially in rural areas.

## Conclusion

Early loss to follow-up and failure to initiate treatment was higher among children with drug-resistant TB than among adults in these provinces. This likely led to underestimates of burden of disease, case notifications, and poor outcomes among this population and indicates a missed opportunity to ensure linkage to care for children who already received a diagnosis. Overall, point-of-care diagnostics eliminating patient departure before diagnosis as well as stronger referral and patient follow-up mechanisms are needed to ensure the most vulnerable patients are not lost between diagnosis and treatment.

## Supporting information

S1 Database Joint all source >08<13 MDR. (XLS)
